# Digital and Navigational Health Literacy in Swiss Cancer Survivors Compared With the General Population: Cross-Sectional Questionnaire Study

**DOI:** 10.2196/84228

**Published:** 2026-05-25

**Authors:** Julia Seinsche, Elena Alder, Tami Wehrmann, Jörg Haslbeck, Karin Ribi, Saskia Maria De Gani

**Affiliations:** 1 Careum School of Health, Kalaidos University of Applied Sciences, Gloriastrasse 18a, Zurich, 8006, Switzerland; 2 Careum Center for Health Literacy, Careum Foundation, Pestalozzistrasse 3, Zürich, 8032, Switzerland, 41 43 222 64 29; 3 Institute of Primary Care, University of Zurich, University Hospital Zurich, Zurich, Switzerland

**Keywords:** digital health literacy, navigational health literacy, self-management skills, cancer survivors, health literacy survey, Switzerland

## Abstract

**Background:**

The number of cancer survivors in Switzerland is increasing. Many individuals face challenges in dealing with health-related information and navigating the health care system. Digitalization offers new care and support opportunities, but its effective use requires digital and navigational health literacy (HL) and self-management skills.

**Objective:**

This study aimed to assess the competencies of cancer survivors in Switzerland, compare them with those of the general population, and identify vulnerable subgroups.

**Methods:**

A cross-sectional online survey was conducted using validated scales from the Health Literacy Survey 2019‐2021 (HLS_19_), measuring digital HL, interaction with digital devices, frequency of use of digital resources, and navigational HL. Self-management skills were assessed with the Health Education Impact Questionnaire (heiQ). HLS_19_ scores were compared to data from the general Swiss population and a subpopulation with chronic diseases provided by the Federal Office of Public Health, using Mann-Whitney *U* tests, chi-square tests, and independent *t* tests. Regression analyses identified associations of sociodemographic and clinical factors with digital and navigational HL and self-management skills.

**Results:**

A total of 131 cancer survivors (74.8% female, 41% with breast cancer) completed the survey. Only 30% reported high digital HL, while self-management skills were generally high, particularly in “health service navigation” (median 3.6, IQR 0.8 on a 4-point Likert scale) and “constructive attitudes” (median 3.6, IQR 1). Compared to the general population, cancer survivors had significantly higher scores in digital interaction and digital resource use (*P*≤.002), while no differences were observed in digital or navigational HL. Digital and navigational HL, as well as self-management, were consistently associated with education level, social support, and financial deprivation.

**Conclusions:**

Cancer survivors in Switzerland report low digital and navigational HL, comparable to the general population, while self-management skills were strong overall. Support strategies should simultaneously target individual and systemic factors to improve cancer survivors’ ability to access and use digital health resources. Future research should include a more representative sample to validate these findings.

## Introduction

The number of cancer survivors is increasing worldwide due to a combination of factors, including population growth, aging, and improvements in cancer survival [1,2]. In Switzerland, there were approximately 210,350 cancer survivors in 2017 [3], and the Swiss Cancer League estimated that this number will increase to approximately 450,000 in 2022 [4]. The journey of cancer survivors can be characterized by different phases, including the acute phase involving diagnosis and treatment, the transitional phase following successful treatment completion, the extended phase, in which cancer survivors live with cancer as a chronic disease or undergo ongoing treatment, and the cancer- and treatment-free phase, in which late and long-term health and psychological effects may occur [5,6].

Cancer survivors face several challenges throughout these phases, including dealing with a wide range of health-related information and navigating an increasingly complex health and social care system. They are expected to understand complex information about their diagnosis, learn new medical terminology, take an active role in decision-making regarding treatment and health service options, adhere to treatment plans, assess and manage symptoms, and adopt health-promoting behaviors [7,8]. These challenges are reflected in the priorities reported by cancer survivors, who expressed a desire to better understand their illness’s chronicity, learn how to live with cancer, receive information and resources for care and self-management, and be empowered and engaged in their care [9]. The guidelines and models of survivorship care emphasize the importance of supporting cancer survivors’ self-management skills to address the medical and emotional consequences of disease and treatment and optimize health and recovery across the cancer journey [6,9-11]. Moreover, self-management is recognized as an essential aspect of general health literacy (HL) in cancer care [9]. HL entails people’s knowledge, motivation, and competencies to access, understand, appraise, and apply health information to make judgments and take decisions in everyday life concerning health care, disease prevention, and health promotion to maintain or improve quality of life [12].

With the rapid digital transformation of health care, an increasing number of technology-enabled solutions are being implemented in cancer care [13], providing new opportunities to support cancer survivors and their self-management [14-17]. As digital solutions become more integrated into cancer care, digital HL—the ability to find, understand, evaluate, and use health information from digital sources [18,19]—is becoming increasingly important for cancer survivors’ HL. Digital HL enables them to benefit fully from these innovations and manage their care effectively. However, an international report indicates that the general population in many European countries has only moderate levels of digital HL. Notably, the Swiss participants reported particularly low competencies. The corresponding survey conducted in Switzerland found that 72% (1801/2502) of respondents reported difficulties in dealing with digital health-related information to a degree that indicates a widespread low level of digital HL [20]. Furthermore, even lower digital HL scores were observed in those enduring one or more chronic diseases compared with individuals without chronic illnesses [20].

In addition, despite the advances in the digitalization of health care, the Swiss health system remains fragmented and is characterized by insufficient coordination and communication among the different care providers [21,22]. The ability to navigate a health system is described as navigational HL. Specifically, it encompasses the knowledge, motivation, and skills to deal with information for navigating health care systems and services to obtain the most appropriate health care [20,23]. Navigational HL is notably low in most European countries, especially compared to other domains of HL. On average, 45% of the respondents rated navigation tasks as “difficult” or “very difficult.” In Switzerland, 74% of the population reported low navigational HL. Individuals living with one or more chronic conditions found it even more difficult to use information from their doctor to manage their condition and reported lower navigational HL than those without chronic conditions [20].

Although the importance of self-management support in survivorship care has been increasingly emphasized [10], digital and navigational HL have not yet been explicitly addressed in survivorship care. Furthermore, there is a paucity of data on digital and navigational HL and self-management skills among cancer survivors in Switzerland.

To address this gap, the primary objective of this study was to assess digital and navigational HL as well as self-management skills among adult cancer survivors in Switzerland, and to compare their levels of digital and navigational HL with those of the general Swiss population.

The secondary objective was to examine the associations between sociodemographic and clinical factors and digital and navigational HL as well as self-management skills.

## Methods

### Study Design and Procedures

A cross-sectional online survey was conducted to assess digital and navigational HL and self-management skills in adult cancer survivors in Switzerland using nonprobabilistic sampling. To reach cancer survivors in different phases of their disease, a self-declared cancer diagnosis was the only criterion to participate in the survey. Recruitment included advertising the survey at several time points with flyers at health and cancer care institutions, as well as through corporate social media channels (eg, LinkedIn [LinkedIn Corp] and Instagram [Meta Platforms]), especially to minimize the effort required for recruitment by health and social care staff. No preselection of cancer survivors occurred to avoid the screening burden at the supporting institutions. To reach potential participants who preferred analog measures, printed questionnaires were also provided to the recruiting institutions.

Participants completed the survey online (or by paper and pencil, if preferred) using a web-based survey tool (UNIPARK; Tivian XI GmbH) between June and December 2024. The survey was available in German and French, as these are major languages spoken in Switzerland.

### Ethical Considerations

The study was submitted to the ethics committee of the Canton of Zurich, which confirmed that the study does not fall under the Human Research Act and therefore granted a waiver (Req-2024‐00298). The study complies with the principles of the Declaration of Helsinki.

The advertisement for participation included initial information about the study, while more detailed information on the study aim, expected survey completion time, data protection, and anonymity of collected data were provided before starting the survey. After reading this information, participants were asked to confirm their consent to participate.

### Measures

The online survey consisted of four parts, including (1) sociodemographic information, (2) questions on digital HL, (3) navigational HL, and (4) self-management skills.

#### Sociodemographic Information

Sociodemographic characteristics (age, gender, living situation, education based on International Standard Classification of Education [ISCED] levels, experience as a health care professional, place of birth, and mother tongue) were collected alongside clinical characteristics (cancer type, date of diagnosis, and other chronic diseases) and the use of cancer care services.

Additionally, 2 indices were calculated [24]. First, a financial deprivation index, which is based on the following questions answered on a 5-point Likert scale: how easy or difficult it is to (1) afford medication, if needed, (2) afford medical examinations and treatments, if needed, and (3) pay bills at the end of the month. The score was defined as the percentage of items answered with “very difficult” or “difficult” and operationalized into four categories (1) none=0%, (2) some=33.33%, (3) considerable=66.67%, and (4) severe=100%.

Second, a social support index was created based on the Oslo Social Support Scale (OSSS-3) [25]. This scale comprises three items: (1) the number of close and reliable persons (1=“no person”-4=“6 or more”), (2) interest and concern of other people regarding the participant’s life (1=“none”-5=“a lot”), and (3) difficulty to get practical help from neighbors (1=“very difficult”-5=“very easy”). A sum score was calculated, ranging between 3 and 14, with a higher score corresponding to a high amount of social support. Following Kocalevent et al [25], the score was operationalized into three categories (1) 3‐8=poor social support, (2) 9‐11=moderate social support, and (3) 12‐14=strong social support.

#### Digital and Navigational HLs

##### Overview

To assess digital and navigational HL, the HLS_19_-DIGI (Health Literacy Survey 2019-2021–Digital Health Literacy) [19,20] and the HLS_19_-NAV (Health Literacy Survey 2019-2021–Navigational Health Literacy) [26,27] were used as part of the European Health Literacy Survey 2019‐2021 (HLS_19_) of the World Health Organization (WHO) Action Network Measuring Population and Organizational Health Literacy (M-POHL). The HLS_19_-DIGI and the HLS_19_-NAV indices were constructed following the recommendations of the HLS_19_ Consortium of the WHO Action Network M-POHL [24]. This consortium validated the survey used in each country, including the translations used in the HLS_19-21_-CH study and this study. The instruments were validated by Cronbach α for reliability, confirmatory factor analysis, and unidimensionality by Rasch analyses. In Switzerland, both instruments showed high reliability (HLS_19_-DIGI Cronbach α=0.85; HLS_19_-NAV Cronbach α=0.88) [27].

##### Digital HL

The HLS_19_-DIGI consists of three parts:

1. The 8-item HLS_19_-Digital Health Information (HLS_19_-DIGI-HI) scale, which assesses the ability to deal with digital health information,
2. The 2-item HLS_19_-Digital Interaction (HLS_19_-DIGI-INT) scale, focusing on the interaction with digital resources for health, and
3. The 6-item HLS_19_-Digital Device (HLS_19_-DIGI-DD) scale measures the frequency of digital resource usage.

Items of the first 2 parts (HLS_19_-DIGI-HI and HLS_19_-DIGI-INT) were operationalized by measuring the perceived difficulty of tasks related to digital health information and resources using a 4-point Likert scale, ranging from “very easy” to “very difficult” [24]. Only participants with >80% (HLS_19_-DIGI-HI) or 100% (HLS_19_-DIGI-INT) valid responses (invalid=“I don’t know” or missing values) were included in all analyses. The respective scores were calculated as the mean of the items’ numeric values, scaled from 0 to 100, following the rules proposed by M-POHL. Higher scores indicate a higher level of digital HL [24].

To gain deeper insights into the participants’ responses, an index [28] was calculated based on the HLS_19_-DIGI-HI results. This score is defined as the percentage of items with valid responses that were answered with ”very easy” or “easy.” Additionally, for easier comprehensibility, the following categories were created.

1. Category excellent: percentage of “very easy” items ≥50%” AND “(very) difficult”<8.334%;
2. Category sufficient: percentage of “(very) easy” items >83.33%;
3. Category inadequate: percentage of “very easy” items <8.334% AND “(very) difficult” ≥50%;
4. Category problematic: all cases which do not fall into one of the other 3 categories.

Participants assigned to the categories “excellent” and “sufficient” were classified as having high digital HL, while those in the “inadequate” and “problematic” categories were classified as having low digital HL [24].

The HLS_19_-DIGI-DD (frequency of usage) response options range from 1 “not relevant for me or less than once a week” to 5 “more than once per day” in a typical week [28]. As in the HLS_19_-DIGI-HI scale, only participants with >80% valid responses were included in the analyses. A mean score was calculated as the relative measure for the frequency of use of health-related digital resources. This mean value has no direct interpretation but was used as a potential determinant of digital HL.

##### Navigational HL

The 12-item HLS_19_-NAV [26,27] is a self-report questionnaire that evaluates the ability to access, understand, appraise, and apply information on navigational issues, using a 4-point Likert scale ranging from “very easy” to “very difficult.” The same response options and score calculations as for the HLS_19_-DIGI-HI apply, including the analysis of an index score and navigational HL categories. Participants with fewer than 80% valid items were excluded from the analysis. Higher scores indicate a higher level of navigational HL.

### Self-Management Skills

Self-management skills were assessed using the Health Education Impact Questionnaire (heiQ). The original self-report questionnaire includes 42 items grouped into 8 dimensions to measure the benefits of patient education programs across chronic conditions [29]. Of these 8 heiQ dimensions, five have been identified as critical dimensions to empower patients with cancer: (1) emotional distress (heiQ3), (2) constructive attitudes and approaches (heiQ5), (3) skill and technique acquisition (heiQ6), (4) social integration and support (heiQ7), and (5) health service navigation [30]. Therefore, for this study, a cancer-specific 25-item version including only these 5 dimensions was used [31,32]. Each question is answered on a 4-point Likert scale ranging from 1 (“strongly disagree”) to 4 (“strongly agree”), except for the emotional distress scale, which is scored in reverse. A mean score for each scale was calculated, with higher scores indicating better self-management skills. The heiQ has shown good psychometric properties [31,32].

### Statistical Analyses

#### Sample Size

The sample size calculation was based on the expected proportion of people reporting low digital and navigational HL. For cross-sectional surveys to represent a proportion, the formula for a qualitative variable applies [33]. Different scenarios for the calculation were used, setting the proportion of people with low digital HL to 72% according to the reference of the Swiss population [20] and to 39% or 66%, respectively, according to 2 studies in patients with cancer [34,35], with a 95% CI (*z*=1.96), and the absolute error set to 5%. This led to a desired sample size between 310 and 366 participants.

#### Data Analysis

Data were analyzed with SPSS (version 27; IBM Corp). Descriptive statistics were calculated for all sociodemographic variables, HL, and self-management measures. To investigate whether digital HL was associated with the frequency of use of digital devices and resources, a Spearman correlation analysis was performed using the scaled mean values HLS_19_-DIGI-HI and HLS_19_-DIGI-INT and the HLS_19_-DIGI-DD mean value. Differences between the study sample and the Swiss general population (SGP), as well as a subgroup with chronic conditions, were analyzed using Mann-Whitney *U* tests, chi-square tests, and independent *t* tests. Concerning the latter, the Welch test was interpreted in case of homogeneity of variances was violated. As gender distribution differed between this study’s population and the SGP, comparisons were conducted for the whole sample as well as stratified by gender. The association of sociodemographic and clinical factors with digital and navigational HL and self-management skills was investigated by means of linear regressions with the scaled mean scores of HLS_19_-DIGI-HI and HLS_19_-NAV, as well as mean values of all heiQ dimensions, as dependent variables and age, gender, education, health care profession, chronic diseases, migration, financial deprivation, and social support as predictors. For all categorical variables (age categories, education, and chronic diseases), dummy variables were created and included in the regression analyses. As this led to a high number of (potentially irrelevant) predictors, single regression analyses were performed, and only those sociodemographic and clinical factors that showed significant associations with the respective independent variables were then included in the joint regression analyses. In all analyses, a *P* value of <.05 was considered significant.

## Results

### Sociodemographic and Clinical Characteristics

A total of 131 participants completed the survey. Table 1 provides an overview of the sociodemographic and clinical characteristics of the participants.

**Table 1.** Sociodemographic and clinical characteristics of included participants (N=131).

Variables	Frequency, n (%)	
Gender		
Women	98 (74.8)	
Men	32 (24.4)	
Missing	1 (0.8)	
Age group (years)		
30‐39	9 (6.9)	
40‐49	23 (17.6)	
50‐59	46 (35.1)	
60‐69	38 (29.0)	
≥70	14 (10.7)	
Missing	1 (0.8)	
Highest educational level		
Primary education (ISCED^a^ level 1)	2 (1.5)	
Lower secondary education (ISCED level 2)	38 (29.0)	
Upper secondary education (ISCED level 3)	9 (6.9)	
Post-secondary non-tertiary education (ISCED level 4)	34 (26.0)	
Short-cycle tertiary education (ISCED level 5)	0 (0.0)	
Bachelor (ISCED level 6)	10 (7.6)	
Master or Licentiate (ISCED level 7)	25 (19.1)	
Doctorate (ISCED level 8)	10 (7.6)	
Unknown	3 (2.3)	
Living situation		
With a partner, without children	51 (38.9)	
With a partner and children	27 (20.6)	
Single-parent family	4 (3.1)	
Shared flat	3 (2.3)	
Single-person household	43 (32.8)	
Other type of household (eg, multifamily household)	3 (2.3)	
(Previous) work as a health care professional		
Yes	36 (27.5)	
No	91 (69.5)	
Don’t know	4 (3.1)	
Born in Switzerland		
Yes	102 (77.9)	
No	29 (22.1)	
Mother tongue other than language spoken at the place of residence		
Yes	30 (22.9)	
No	99 (75.6)	
Don’t know or missing	2 (1.5)	
Cancer type		
Pancreatic cancer	3 (2.3)	
Bladder cancer	1 (0.8)	
Breast cancer	53 (40.5)	
Colon cancer	10 (7.6)	
Ovarian or cervical cancer	9 (6.9)	
Skin cancer	7 (5.3)	
Brain tumor or metastases	11 (8.4)	
Testicular cancer	1 (0.8)	
Lymphoma	13 (9.9)	
Liver cancer	3 (2.3)	
Leukemia	4 (3.1)	
Lung cancer	16 (12.2)	
Stomach cancer	1 (0.8)	
Kidney cancer	2 (1.5)	
Prostate cancer	5 (3.8)	
Thyroid cancer	3 (2.3)	
Other	21 (16)	
Other chronic diseases		
None	71 (54.2)	
One	32 (24.4)	
More than one	25 (19.1)	
Don’t know	3 (2.3)	
Financial deprivation		
None	103 (79.8)	
Some	9 (7.0)	
Considerable	7 (5.4)	
Severe	10 (7.8)	
Social support		
Poor	21 (17.0)	
Moderate	48 (39.0)	
Strong	55 (44.0)	

a ISCED: International Standard Classification of Education (8 Levels).

Most participants (53/131, 40.5%) were diagnosed with breast cancer, followed by lung cancer (16/131, 12.2%), lymphoma (13/131, 9.9%), and brain tumors or metastases (11/131, 8.4%). The average time since diagnosis was 6 (SD 5.2) years. Most participants (71/131, 54.2%) did not report any other chronic disease, while 24.4% (32/131) had one and 19.1% (25/131) had multiple chronic diseases. Table 2 displays the number of participants using one or more cancer care services, such as support groups. Complementary medical advice was by far the most frequently sought service (53/131, 40.5%).

**Table 2.** Participation in cancer care services (N=131).

Variable	Frequency, n (%)	
Nursing care consultation	39 (29.8)	
Support groups	25 (19.1)	
Complementary medical advice	53 (40.5)	
Services by patient organizations	25 (19.1)	
None of these services	41 (31.1)	
Don’t know/missing	2 (1.5)	

### Digital HL

The mean digital HL index score, thus the average percentage of “very easy” and “easy” items in the HLS_19_-DIGI-HI, was 57.8 (SD 35.4), with 6.6% (8/122) of the participants having excellent, 30.3% (37/122) sufficient, 36.9% (45/122) inadequate, and 32.8% (40/122) problematic digital HL. Based on these categories, 30.3% (37/122) of participants were classified as having high digital HL and 69.7% (85/122) having low digital HL. The scaled mean score of all participants was 52.88 (SD 20.21), while those with high digital HL had a mean score of 74.77 (SD 10.93).

**Figure 1.**
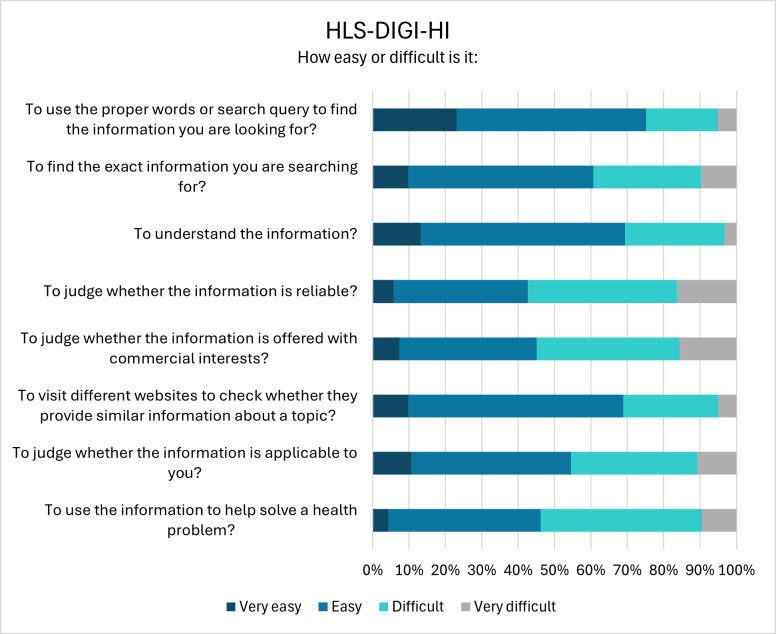
Ability to deal with digital health information (HLS_19_-DIGI-HI). Note that the response option “I don’t know” is not displayed since it is considered an invalid response.

Looking at the individual items of the HLS_19_-DIGI-HI (Figure 1), it becomes apparent that the majority struggled with judging whether information is reliable (70/122, 57.40%) or offered with commercial interest (67/122, 54.90%) and to use the information to help solve a health problem (63/117, 53.80%). On the other hand, most participants found it easy to use proper search terms (91/121, 75.20%), understand health-related online information (84/121, 69.40%), and compare the information provided by different websites (84/122, 68.80%). It should be noted that those who responded “I don’t know” are not included in the reported percentages, as these responses are considered invalid.

Regarding the interaction with digital resources, the HLS_19_-DIGI-INT scaled mean score for all participants in this study was 62.86 (SD 27.30). Most participants found it easy to express their opinion in social media forums (65/92, 70.6%) and to formulate a written question for a health care provider (62/92, 67.4%; Multimedia Appendix 1). Notably, 39 out of 131 (29%) participants responded with “I don’t know” to either one or both items and were, thus, considered invalid.

In the HLS_19_-DIGI-DD, participants’ mean score was 1.64 (SD 0.67), meaning that on average, participants used digital resources 1‐3 times per week. Digital devices such as pedometers or smartwatches and health apps were used most frequently, with 25.4% (32/126) or 25.2% (32/127), respectively, using them once or more times a day. Contrary to this, social media and digital interactions with the health system, for instance, for online appointments, were used less than once per week or were considered irrelevant by 77.0% (97/126) and 85.7% (108/126), respectively (Multimedia Appendix 2).

The correlation analyses did not reveal any significant correlations of the mean frequency of use of digital devices and resources (HLS_19_-DIGI-DD) with either the scaled mean scores of HLS_19_-DIGI-HI (Spearman ρ=0.06; *P*=.54) and HLS_19_-DIGI-INT (Spearman ρ=0.09; *P*=.38) or with any of the subitems (Spearman ρ=0.012; *P*=.89; Spearman ρ=0.11; *P*=.32).

### Navigational HL

In the HLS_19_-NAV, participants had a mean navigational HL index score of 53.78 (SD 30.89), with 4.8% (6/124) of the participants categorized as having excellent, 24.2% (30/124) sufficient, 54.8% (68/124) inadequate, and 21% (26/124) problematic navigational HL. Based on these categories, 24.2% (30/124) of participants were classified as having high navigational HL, and overall, participants had a scaled mean score of 51.3 (SD 18.08) points.

**Figure 2.**
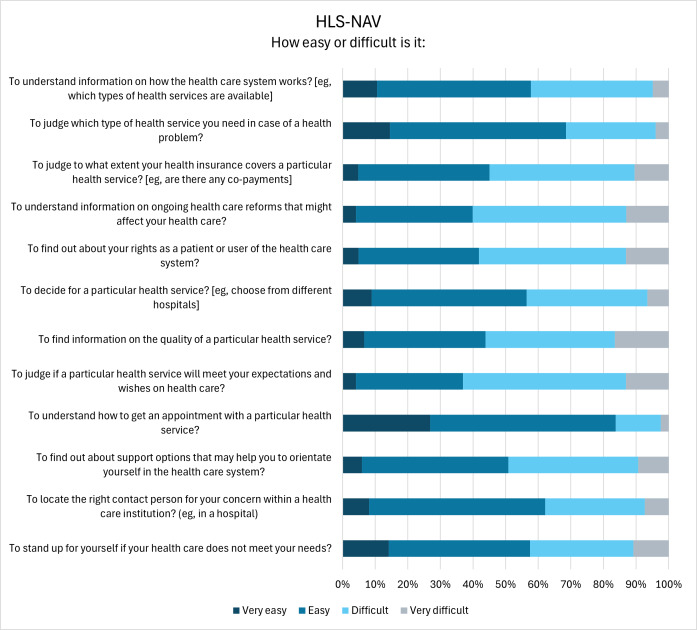
Level of difficulty of different navigational health literacy (HLS_19_-NAV) actions. The response option “I don’t know” is not displayed since it is considered an invalid response.

Most participants struggled with judging whether a particular health service will meet their expectations (77/122, 63.1%), understanding information on ongoing health care reforms that might affect their health (74/123, 60.2%), and finding out about their rights as a patient (71/122, 58.2%; Figure 2). On the other hand, the majority found it easy to judge which type of health service they need (85/124, 68.5%) and to understand how to get an appointment (103/123, 83.7%).

### Comparison With the General Swiss Population

Comparing these results with raw data from the HLS_19-21_ study investigating the general adult population living in Switzerland (SGP), the present study’s population was significantly older (*U*=128055.00; *P*<.001) and differed in gender distribution (*χ*²_1_=28.70; *P*<.001; *φ*=.10), with a higher proportion of female participants compared to the SGP. No significant differences were found in the ability to deal with digital health information (HLS_19_-DIGI-HI; *P*=.64). In contrast, both men and women in this study reported a significantly higher ability to interact with digital resources (HLS_19_-DIGI-INT, *P*<.001 to *P*=.02) and a more frequent use of digital devices and resources (HLS_19_-DIGI-DD, *P*<.001). Navigational HL (HLS_19_-NAV) did not differ significantly between groups (*P*=.87). (Table 3 and Multimedia Appendix 3).

**Table 3.** Differences in digital and navigational health literacy between cancer survivors and the Swiss general population. The subpopulation comprises individuals with one or more chronic conditions.

Dependent variable and SGP^a^ group	SGP (n)	CS^b^ (n)	Test	*t* test (df)	*P* value	MD^c^ (95% CI)			Cohen *d*
Digital HL^d^ (HLS^e^_19_ -DIGI-HI^f^ scaled mean value)									
SGP whole group									
Gender									
All	2359	122	*t* test	–0.46 (2479)	.64	−0.85 (–4.46 to 2.75)			0.04
Men	1165	30	*t* test	–1.03 (1193)	.30	–3.76 (–10.91 to 3.38)			0.19
Women	1191	91	*t* test	–0.09 (1280)	.93	–0.18 (–4.43 to 4.06)			0.01
SGP subpopulation									
Gender									
All	852	122	*t* test	–1.96 (972)	.05	–3.84 (–7.69 to 0.00)			0.19
Men	409	30	*t* test	–1.97 (437)	.05	–7.60 (–15.17 to –0.03)			0.37
Women	442	91	*t* test	–1.00 (531)	.32	–2.31 (–6.87 to 2.24)			0.12
Digital HL (HLS_19_ -DIGI-INT^g^ scaled mean value)									
SGP whole group									
Gender									
All	2156	92	*t* test	–3.10 (2246)	<.01	–8.30 (–13.56 to –3.04)			0.33
Men	1082	20	Welch test	–4.08 (20.4)	<.001	−15.95 (–24.10 to –7.80)			0.66
Women	1071	71	*t* test	–2.32 (1140)	.02	−7.44 (–13.73 to –1.14)			0.28
SGP subpopulation									
Gender									
All	752	92	*t* test	–4.15 (842)	<.001	–12.24 (–18.03 to –6.45)			0.46
Men	365	20	Welch test	–5.02 (24.2)	<.001	–20.48 (–28.90 to –12.06)			0.79
Women	386	71	*t* test	–3.02 (455)	<.01	–10.63 (–17.54 to –3.71)			0.39
Navigational HL (HLS_19_-NAV)^h^									
SGP whole group									
Gender									
All	2478	124	*t* test	–0.17 (2600)	.87	–0.26 (–3.37 to 2.84)			0.02
Men	1214	31	*t* test	–1.56 (1243)	.12	–4.86 (–10.95 to 1.24)			0.28
Women	1261	92	*t* test	0.38 (1351)	.70	0.71 (–2.94 to 4.36)			0.04
SGP subpopulation									
Gender									
All	910	124	*t* test	–1.04 (1032)	.30	–1.67 (–4.83 to 1.48)			0.10
Men	436	31	*t* test	–2.26 (465)	.02	–6.84 (–12.79 to –0.89)			0.42
Women	473	92	*t* test	–0.04 (563)	.97	–0.09 (–3.94 to 3.76)			0.01

a SGP: Swiss general population (data provided by the Federal Office of Public Health).

b CS: cancer survivor.

c MD: mean difference.

d HL: health literacy.

e HLS: Health Literacy Survey.

f HLS_19_-DIGI-HI: Health Literacy Survey 2019-2021 - Digital Health Information subscale.

g HLS_19_-DIGI-INT: Health Literacy Survey 2019-2021–Digital Interaction subscale.

h HLS_19_-NAV: Health Literacy Survey 2019-2021–Navigational Health Literacy.

When compared to the SGP subpopulation of participants with a chronic condition, participants were similar in age (*U*=60785.50; *P*=.92) but differed in gender distribution (*χ*²_1_=24.85; *P*<.001; *φ*=.15). No significant differences were found in digital HL (HLS_19_-DIGI-HI; *P*=.05). However, both male and female participants of this study had higher digital interaction abilities (HLS_19_-DIGI-INT; *P*<.01) and a higher frequency of digital device use (*P*<.001). Concerning navigational HL, a significant difference was found only among men (*P*<.02).

Overall, male and female participants of this study showed a similar pattern when compared with the SGP. However, across all variables, effect sizes were consistently larger among men (Table 3 and Multimedia Appendix 3).

### Self-Management Skills

**Figure 3.**
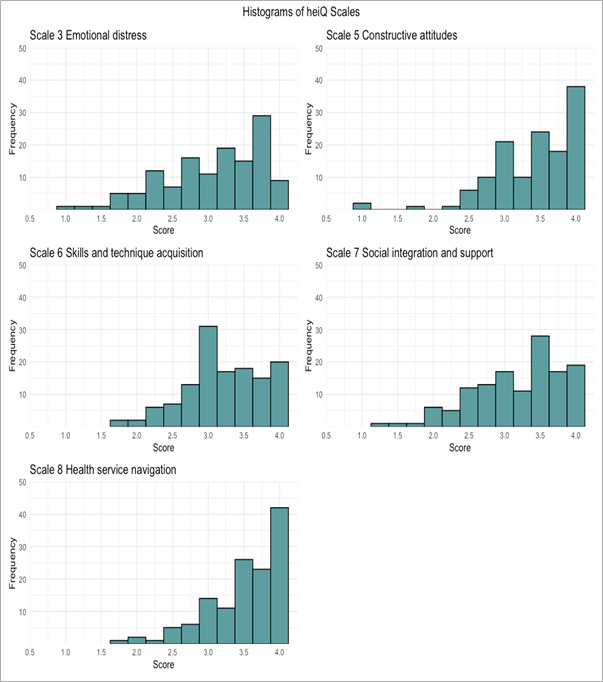
Histograms of Health Education Impact Questionnaire (heiQ) scales. The higher scores indicate better self-management skills; thus, the emotional distress scale was reverse scored so that higher values indicate lower emotional distress.

Figure 3 shows that in all subscales, most participants indicated high self-management, with the highest scores in “health service navigation” (median 3.6, IQR 0.8) and “constructive attitudes” (median 3.6, IQR 1) and the lowest in “emotional distress” (median 3.2, IQR 1).

### Associations of Sociodemographic and Clinical Factors With Digital and Navigational HL and Self-Management Skills

#### Digital HL

The single regression analyses revealed significant associations between digital HL (HLS_19_-DIGI-HI) and financial deprivation (B=−.154; *P*=.01), being a health care professional (B=−9.37; *P*=.02), having the highest level of education (ISCED level 8; B=20.74; *P*=.002), and social support (B=2.30; *P*=.004). Gender, age, migration background, time since diagnosis, and having a chronic illness other than cancer did not show significant associations with digital HL in the single regression analyses. The joint regression model (Regression model 1, Table 4) was statistically significant (*P*<.001) with an adjusted *R*^2^ value of .16, indicative of a moderate/medium goodness-of-fit according to Cohen (1988). In this model, ISCED level 8 (B=19.36; *P*=.003) as well as the level of social support (B=2.11; *P*=.02) had significantly positive effects on digital HL. The standardized coefficients show that education was the strongest predictor in the model (*β*=0.28).

**Table 4.** Regression model 1. The dependent variable in this regression model is the scaled mean score of Health Literacy Survey 2019-2021–Digital Health Information (HLS_19_*-*DIGI-HI).

Predictor	B^a^ (95% CI)	SE	Standardized *β* coefficient	*t* test (df)	*P* value
Constant	34.25 (7.04 to 61.47)	13.73	—^b^	2.50 (106)	.01
Financial deprivation	–.06 (–0.19 to 0.07)	0.07	–0.09	–0.92 (106)	.36
Health care professional	3.14 (–5.05 to 11.34)	4.13	0.07	0.76 (106)	.45
ISCED 8^c^	19.36 (6.65 to 32.07)	6.41	0.28	3.02 (106)	.003
Social support	2.11 (0.37 to 3.85)	0.88	0.24	2.41 (106)	.02

a B: regression coefficient.

b Not applicable.

c ISCED 8: International Standard Classification of Education, 8 levels.

#### Navigational HL

In the single regression analyses, navigational HL was significantly associated with ISCED levels 1 (B=−33.93; *P*=.01) and 8 (B=14.67; *P*=.01), financial deprivation (B=−.24; *P*<.001), being born in Switzerland (B=−11.25; *P*=.004), having no other chronic disease (B=10.08; *P*=.002), and social support (B=2.60; *P*<.001). However, no significant associations with gender, age, time since diagnosis, and having worked as a health care professional were found. The joint regression model (Regression model 2, Table 5) was statistically significant (*P*<.001) and explained a high amount of variance (*R*^2^=.29). In this model, only financial deprivation (B=−.133; *P*=.03) and being born in Switzerland (B=−11.47; *P*=.003) were significantly associated with navigational HL. The standardized coefficients show that being born in Switzerland was the strongest predictor in the model (*β*=0.248).

**Table 5.** Regression model 2.Note. Dependent variable: scaled mean score of Health Literacy Survey 2019-2021–Navigational Health Literacy (HLS_19_*-*NAV).

Predictor	B^a^ (95% CI)	SE	Standardized *β* coefficient	*t* test (df)	*P*-value
Constant	42.69 (24.13 to 61.47)	9.36	—^b^	4.56 (106)	<.001
ISCED^c^ 1	–22.43 (–45.70 to 0.07)	11.73	–0.16	–1.91 (106)	.06
ISCED 8^c^	9.18 (–1.55 to 11.34)	5.41	0.14	1.70 (106)	.09
Financial deprivation	–.13 (–0.25 to –0.01)	0.06	–0.21	–2.21 (106)	.03
Born in Switzerland	–11.47 (–18.95 to –4.0)	3.77	–0.25	–3.04 (106)	.003
No chronic diseases	5.77 (–0.47 to 12.01)	3.15	0.15	1.83 (106)	.07
Social support	1.45 (–0.04 to 2.95)	0.75	0.18	1.93 (106)	.06

a B: regression coefficient

b Not applicable.

c ISCED: International Standard Classification of Education (8 levels; level 1=primary education–level 8=doctorate).

### Self-Management Skills

The regression analyses of the heiQ subscales revealed that three variables significantly influenced the different dimensions of self-management skills: (1) financial deprivation (*P*<.001-.03); (2) the lowest level of education (ISCED 1; *P*=.002-.04); and (3) social support (*P*≤.001-.005) (Multimedia Appendix 4). Only in heiQ6, the time since diagnosis was another significant predictor (*P*=.004). All regression models were statistically significant with *P*<.001 and adjusted *R*^2^ values ranged from *R*^2^=.25 (heiQ3) to *R*^2^=.46 (heiQ7); thus, the models explain a medium to high amount of variation.

## Discussion

### Principal Findings

Sufficient digital HL, navigational HL, and self-management skills are essential for cancer survivors to successfully manage the many challenges they encounter throughout their cancer journey. The present study delivers first-time quantitative data about digital and navigational HL of cancer survivors in Switzerland in comparison to the general Swiss population and describes their self-management skills.

The results indicate that a substantial proportion of cancer survivors in Switzerland reported difficulties in managing digital health information and services (69.7%) and in successfully navigating the health care system (75.8%), reflecting generally low levels of digital and navigational HL. Previous studies have yielded inconsistent results regarding digital HL among patients with cancer. For instance, a Canadian study found that 66.3% of patients with lung cancer perceived themselves as having low digital HL [35], whereas other studies reported lower proportions (41.5% [36] and 39.4% [34], respectively) among patients with various cancer types. These discrepancies may partly be explained by the use of different instruments to assess digital HL. In addition, cross-country variations in health care systems and the support provided to cancer survivors by health care professionals and community networks may further contribute to such differences.

In comparison with the general adult population living in Switzerland [20], a slightly lower proportion of cancer survivors in this study reported low digital HL levels, while the proportion of those with low navigational HL was slightly higher. However, statistically significant differences were only found for the ability to interact with digital resources, with cancer survivors reporting higher abilities. The latter finding is unexpected given the increased vulnerability of cancer survivors, their significantly higher age, and the negative correlation between digital HL and age, which previous studies observed in both the general population and cancer survivors [20,37,38]. One possible explanation for why cancer survivors still show similar or even higher levels of digital and navigational HL is that they need to interact with the health care system more frequently than the general population, and in recent years, these interactions have increasingly taken place through digital channels. In line with this assumption, cancer survivors reported a significantly higher use of digital health resources than the general population. Although no significant association between digital resource use and digital HL was found, the combination of frequent engagement with health care services and digital tools may nonetheless contribute to the development of certain digital and navigational HL skills, irrespective of age. In addition, a high proportion of participants reported using complementary medical advice as well as specialist or advanced practice nurse consultations. Previous research suggests that support from health care professionals [39]—such as specialized breast or lung cancer care nurses available to patients with cancer in Switzerland [40-42]—as well as informational and emotional support from health communities [43] may enhance digital HL of cancer survivors by helping them find, understand, appraise, and apply relevant digital health information. Despite these potential facilitating factors, the large proportion of cancer survivors with low digital and navigational HL observed in this study indicates that access to such services may be limited in Switzerland or that existing services may still be insufficiently tailored to cancer survivors’ digital HL needs. This interpretation is consistent with previous research showing that difficulties in accessing coordinated, personalized services and care are common rather than exceptional [44].

Beyond the overall HLS_19_ mean scores, an item-level analysis of individual questionnaire items further highlights critical challenges and the importance of support services and improving digital and navigational HL. Cancer survivors reported most difficulties in judging whether the digital information is reliable or offered with commercial interests, and more than half of respondents also indicated difficulties in applying digital information to solve a health problem. These challenges may partly reflect the complexity and individual nature of cancer, as well as the continuous opportunities for new treatment options provided by clinical research. However, similar trends were also observed in the Swiss population study, with 66% of Swiss residents reporting difficulties in assessing the reliability of health information, 63% struggling with determining whether commercial interests were influencing the information, and 53% finding it challenging to apply the information to address a health-related problem [20]. These findings highlight that such difficulties appear to affect people in general, regardless of any specific health condition, and that they may stem from the vast amount of health information available online. This so-called “infodemic”—an overabundance of both accurate health information and mis- or disinformation [45,46]—makes it highly challenging to evaluate the quality, that is, the reliability and the credibility of the information. The spread of mis- and disinformation especially occurs on social media. A study conducted during the COVID-19 pandemic reported that individuals residing in Switzerland did not regard social media as a trustworthy source of health information [47]. This perception may partly explain why most participants in the present study reported using social media for health-related information fewer than once a week.

In addition to improving digital and navigational HL, self-management promotion is recognized as an important strategy to support cancer survivors in dealing with their disease in their everyday life and maintaining and enhancing their health and well-being [10,11]. In this study, most participants reported good self-management skills, consistent with findings from a previous Swiss study of breast cancer survivors [48]. In contrast, the Health Literacy Survey 2019-2021–Switzerland (HLS_19-21_-CH) study [20] found that 66% (613/928) of participants with one or more chronic diseases had low self-management skills. These differences may partly be explained by the instruments used. While the HLS_19-21_-CH study assessed self-management skills with only 4 questions and with a focus on health care–related topics, the heiQ covers topics going beyond, capturing a broader range of self-management dimensions. The overall high level of self-management skills observed in this sample may be explained by the participants’ relatively high level of social support and low levels of financial deprivation. These factors were identified as significant predictors of self-management skills in this analysis. Another explanation may be the fact that self-management focuses on specific behaviors, and informal and practice-based self-management support, such as counseling by health care professionals, nursing consultations, and support groups, addressing the dimensions assessed in our study, may already be relatively well integrated into comprehensive cancer care. However, while various self-management interventions and programs exist internationally, which are expected to foster self-management skills in cancer survivors [49], structured and theory-based self-management interventions and programs have so far been only partially integrated into routine clinical care in Switzerland, with little data on their effectiveness. Available interventions include a peer-led self-management program for breast cancer survivors [48], nurse-led basic symptom self-management support for patients receiving first-line systemic outpatient anticancer treatment [50], and a psycho-educational cancer pain self-management intervention [51]. Although these interventions were generally perceived as beneficial, their broader implementation remains limited due to challenges related to delivery, feasibility, and individual preferences regarding timing and format [48,50].

In contrast, measures that specifically promote digital and navigational HL are still rarely used in practice [52]. This imbalance may explain why self-management skills appear relatively strong in this sample, while digital and navigational HL levels remain low. Nevertheless, the high heiQ scores, particularly on the “health service navigation” scale, appear somewhat surprising given the low scores in digital and navigational HL. This discrepancy can be explained by differences in constructs. Although self-management is recognized as an essential aspect of general HL [9], the instruments used to assess self-management skills and navigational HL are based on different constructs. The “health service navigation” items from the heiQ focus more broadly on relationships and communication with health care professionals, while HLS_19_-NAV items revolve around specific navigational tasks within the entire health system. The lowest self-management skills were observed in relation to emotional distress, which aligns with previous research indicating that emotional needs are among the most frequently unmet needs in patients with breast cancer [53]. This finding underscores the importance of cancer care services addressing not only the physical but also the emotional challenges faced by cancer survivors.

Overall, the findings suggest that efforts to strengthen digital and navigational HL among cancer survivors in Switzerland are needed and that particular attention should be paid to those who experience financial deprivation, have a migration background, lower educational levels, and limited social support. These findings align with previous research that has shown that economic status and HL contribute to inequalities in cancer care experiences in Switzerland [54], suggesting that this may also be true for digital and navigational HL. Moreover, findings are supported by recently published consensus recommendations for comprehensive survivorship care, emphasizing patient empowerment, including the promotion of HL and self-management, as well as psychosocial and socioeconomic support and the involvement of interprofessional experts such as social workers. As a core element for the integration of different types of support, a survivorship plan is recommended [55]. In addition, previous research indicates that interventions, programs, and strategies aimed at strengthening digital and navigational HL of cancer survivors should not only focus on individual competencies. Instead, a more holistic approach is needed, including professional and organizational HL [56]. In addition to individual factors, addressing social, situational, and systemic factors can not only strengthen HL of chronically ill individuals but also reduce burdens and demands placed on them [20,23].

### Limitations

This study has several limitations. First, the target sample size could not be reached. With the smaller than expected sample size, the proportion of cancer survivors with low digital and navigational HL may not be adequately reflected. Differences in methodology may limit the comparability of our sample with the Swiss population. Although the modes of administration were quite similar (primarily online), the recruitment approach differed substantially. In the HLS_19-21_-CH study, participants were recruited via the Swiss Federal Statistical Office’s sampling frame and contacted at their postal address. This sampling method could not be used for the present study, as no information about specific chronic conditions was available. Although opting for a broad recruitment approach, it allowed little control over who would be reached by these strategies. Following the outreach to partner organizations and the launch of social media advertisements, the research team noted an increase in survey engagement. Nevertheless, cancer survivors without ties to these institutions or lacking social media access may have missed the chance to participate. Though time-efficient for professionals, this impersonal recruitment strategy may have limited the number of participants compared to more personalized methods involving direct health care provider contact. Second, sampling bias cannot be ruled out, as the participants were predominantly female and breast cancer survivors. The latter, however, reflects the prevalence of cancer in Switzerland, where breast cancer is the most common cancer diagnosis among women [3] and the most often studied cancer type in the field of HL [56]. Furthermore, a considerable number had professional experience in health care, and the survey was conducted predominantly online. Thus, it was completed primarily by people who have sufficient digital skills and/or feel at least comfortable enough to engage with an online survey. In addition, data from the survey software suggest that the length of the survey or the lack of incentives may have demotivated some respondents from completing the survey. Overall, engagement with the landing page of the survey (around 800 clicks) was significantly higher than the number of people who started the survey (n=250), of which more than half ultimately finished it. Cancer survivors might be already burdened by fatigue or other health-related issues affecting their willingness to participate in or finish such a survey. Taken together, these factors limit the representativeness of the study population for Swiss cancer survivors, limiting the generalizability of the results as well as the comparisons with the HL study in the Swiss population that used a representative sample for the adult residents in Switzerland. Third, the instruments used rely on self-reported ability to perform certain tasks related to digital and navigational HL as well as self-management skills. Although self-report instruments have the advantage that they are easy to complete, often answered more honestly, and well accepted by respondents, they are limited by the fact that participants might misestimate their level of HL and self-management skills [57]. However, objective HL measurements are scarce and untenable in population-based studies [58]. Additionally, all instruments applied in this study were standardized and validated.

### Conclusions

Cancer survivors in Switzerland generally reported low levels of digital and navigational HL, comparable to those of the general Swiss population and subgroups with chronic conditions. These findings emphasize the need to support cancer survivors in Switzerland in dealing with digital health information and navigating the health care system. Particular attention should be given to tasks perceived as the most difficult, including judging the reliability and relevance of health-related information and services, using this information appropriately, and gaining knowledge and understanding of health care reforms and patient rights. Interventions should be better integrated in existing support, targeting digital and navigational HL as well as self-management skills, while considering individual, social, and situational factors, which showed significant associations with these competencies in this study. Given the limitations of this study, future research should examine digital and navigational HL in a larger sample that more accurately reflects the gender distribution and digital affinity of the population. Nevertheless, this study provides valuable insights into digital and navigational HL among cancer survivors in Switzerland. The findings presented here will be complemented with qualitative interview data from cancer survivors and insights from workshops involving cancer survivors and cancer care professionals. These contributions will be used to develop more explicit intervention recommendations.

## Supplementary material

10.2196/84228 Multimedia Appendix 1 Level of difficulty in interacting with digital resources Health Literacy Survey 2019-2021–Digital Interaction (HLS19-DIGI-INT).

10.2196/84228 Multimedia Appendix 2 Frequency of use of digital health devices and resources Health Literacy Survey 2019-2021–Digital Device (HLS19-DIGI-DD).

10.2196/84228 Multimedia Appendix 3 Differences in frequency of digital device use between cancer survivors and the general Swiss population (Mann-Whitney *U* Test).

10.2196/84228 Multimedia Appendix 4 Regression analyses with all significant sociodemographic variables as independent and Health Education Impact Questionnaire (heiQ) subscales as dependent variables.

## Abbreviations

heiQ: Health Education Impact Questionnaire
HL: health literacy
HLS_19_: Health Literacy Survey 2019‐2021
HLS_19-21_-CH: Health Literacy Survey 2019-2021–Switzerland
HLS_19_-DIGI: Health Literacy Survey 2019-2021–Digital Health Literacy
HLS_19_-DIGI-DD: Health Literacy Survey 2019-2021–Digital Device
HLS_19_-DIGI-HI: Health Literacy Survey 2019-2021–Digital Health Information
HLS_19_-DIGI-INT: Health Literacy Survey 2019-2021–Digital Interaction
HLS_19_-NAV: Health Literacy Survey 2019-2021–Navigational Health Literacy
ISCED: International Standard Classification of Education
M-POHL: Measuring Population and Organizational Health Literacy
OSSS-3: Oslo Social Support Scale
SGP: Swiss general population
WHO: World Health Organization

## References

[1] Miller KD, Nogueira L, Devasia T (2022). Cancer treatment and survivorship statistics, 2022. CA Cancer J Clin.

[2] Siegel RL, Miller KD, Fuchs HE, Jemal A (2022). Cancer statistics, 2022. CA A Cancer J Clinicians.

[3] Cirillo P, Feller A, Hošek M (2021). Schweizerischer Krebsbericht 2021: Stand Und Entwicklungen.

[4] Schweiz K (2024). Krebs in der Schweiz: wichtige Zahlen. Krebsliga Schweiz Ligue Suisse Contre le Cancer Liga Svizzera Contro il Cancro.

[5] Miller K, Merry B, Miller J (2008). Seasons of survivorship revisited. Cancer J.

[6] Vaz-Luis I, Masiero M, Cavaletti G (2022). ESMO expert consensus statements on cancer survivorship: promoting high-quality survivorship care and research in Europe. Ann Oncol.

[7] Holden CE, Wheelwright S, Harle A, Wagland R (2021). The role of health literacy in cancer care: a mixed studies systematic review. PLoS ONE.

[8] Chan RJ, Mayer DK, Koczwara B (2020). Building capacity in cancer nurses to deliver self-management support: a call for action paper. Cancer Nurs.

[9] Mehnert-Theuerkauf A, Esser P (2022). Survivorship-care-programme für Krebspatienten: die Bedeutung von Risikostratifizierung, Selbstmanagement- und Gesundheitskompetenzen im Zeitalter digitaler Versorgung. Bundesgesundheitsbl.

[10] Howell D, Mayer DK, Fielding R (2021). Management of cancer and health after the clinic visit: a call to action for self-management in cancer care. J Natl Cancer Inst.

[11] McCorkle R, Ercolano E, Lazenby M (2011). Self-management: enabling and empowering patients living with cancer as a chronic illness. CA Cancer J Clin.

[12] Sørensen K, Van den Broucke S, Fullam J (2012). Health literacy and public health: a systematic review and integration of definitions and models. BMC Public Health.

[13] Shaffer KM, Turner KL, Siwik C (2023). Digital health and telehealth in cancer care: a scoping review of reviews. Lancet Digit Health.

[14] Ahmed S, LePage K, Benc R (2022). Lessons learned from the implementation of a person-centred digital health platform in cancer care. Curr Oncol.

[15] Chan RJ, Crichton M, Crawford-Williams F (2021). The efficacy, challenges, and facilitators of telemedicine in post-treatment cancer survivorship care: an overview of systematic reviews. Ann Oncol.

[16] Moradian S, Maguire R, Liu G (2023). Promoting self-management and patient activation through eHealth: protocol for a systematic literature review and meta-analysis. JMIR Res Protoc.

[17] van den Hurk CJG, Mols F, Eicher M (2022). A narrative review on the collection and use of electronic patient-reported outcomes in cancer survivorship care with emphasis on symptom monitoring. Curr Oncol.

[18] Norman CD, Skinner HA (2006). eHealth literacy: essential skills for consumer health in a networked world. J Med Internet Res.

[19] Levin-Zamir D, Van den Broucke S, Bíró É (2025). Measuring digital health literacy and its associations with determinants and health outcomes in 13 countries. Front Public Health.

[20] Jaks R, De Gani SM, Bieri U, Kocher JP (2023). Health Literacy Survey Schweiz 2019-2021: im Auftrag des Bundesamtes für Gesundheit. Schlussbericht mit überarbeiteten Empfehlungen. https://www.bag.admin.ch/dam/de/sd-web/zTvqdtp8UAbm/schlussbericht-health-literacy-survey-careum.pdf.

[21] Kaltenborn Z, Paul K, Kirsch JD (2021). Super fragmented: a nationally representative cross-sectional study exploring the fragmentation of inpatient care among super-utilizers. BMC Health Serv Res.

[22] Lomazzi M, Borisch B (2022). Game changer in cancer treatment in Switzerland. J Cancer Policy.

[23] Griese L, Schaeffer D, Berens EM (2023). Navigational health literacy among people with chronic illness. Chronic Illn.

[24] The HLS19 Consortium of the WHO Action Network M-POHL (2021). International report on the methodology, results, and recommendations of the European health literacy population survey 2019-2021 (HLS19) of M-POHL. https://m-pohl.net/Int_Report_methdology_results_recommendations.

[25] Kocalevent RD, Berg L, Beutel ME (2018). Social support in the general population: standardization of the Oslo social support scale (OSSS-3). BMC Psychol.

[26] Griese L, Finbråten HS, Francisco R (2022). HLS_19_-NAV-validation of a new instrument measuring navigational health literacy in eight European countries. Int J Environ Res Public Health.

[27] The HLS19 Consortium of the WHO Action Network M-POHL (2022). The HLS19–NAV Instrument to Measure Navigational Health Literacy.

[28] The HLS19 Consortium of the WHO Action Network M-POHL (2022). The HLS19–DIGI Instrument to Measure Digital Health Literacy.

[29] Osborne RH, Elsworth GR, Whitfield K (2007). The health education impact questionnaire (heiQ): an outcomes and evaluation measure for patient education and self-management interventions for people with chronic conditions. Patient Educ Couns.

[30] Maunsell E, Lauzier S, Brunet J, Pelletier S, Osborne RH, Campbell HS (2014). Health-related empowerment in cancer: validity of scales from the health education impact questionnaire. Cancer.

[31] Bélanger A, Hudon C, Fortin M, Amirall J, Bouhali T, Chouinard MC (2015). Validation of a French-language version of the health education impact Questionnaire (heiQ) among chronic disease patients seen in primary care: a cross-sectional study. Health Qual Life Outcomes.

[32] Schuler M, Musekamp G, Faller H (2013). Assessment of proximal outcomes of self-management programs: translation and psychometric evaluation of a German version of the health education impact Questionnaire (heiQ). Qual Life Res.

[33] Charan J, Biswas T (2013). How to calculate sample size for different study designs in medical research?. Indian J Psychol Med.

[34] Melhem SJ, Nabhani-Gebara S, Kayyali R (2023). Digital trends, digital literacy, and e-Health engagement predictors of breast and colorectal cancer survivors: a population-based cross-sectional survey. Int J Environ Res Public Health.

[35] Milne RA, Puts MTE, Papadakos J (2015). Predictors of high eHealth literacy in primary lung cancer survivors. J Cancer Educ.

[36] Heiman H, Keinki C, Huebner J, Working Group Prevention and Integrative Oncology of the German Cancer Society (2018). EHealth literacy in patients with cancer and their usage of web-based information. J Cancer Res Clin Oncol.

[37] Schaeffer D, Gille S, Berens EM (2023). Digitale gesundheitskompetenz der bevölkerung in Deutschland: ergebnisse des HLS-GER 2. Gesundheitswesen.

[38] Hoogland AI, Mansfield J, Lafranchise EA, Bulls HW, Johnstone PA, Jim HSL (2020). eHealth literacy in older adults with cancer. J Geriatr Oncol.

[39] Hildreth K, Henderson N, Bourne G (2023). Recommendations for improving digital health literacy in patients with cancer receiving care in the deep south. JCO Oncology Practice.

[40] (2025). Fachgruppe breast care nurse (BCN-OPS). Onkologiepflege Schweiz.

[41] Eicher M, Kadmon I, Claassen S (2012). Training breast care nurses throughout Europe: the EONS postbasic curriculum for breast cancer nursing. Eur J Cancer.

[42] Serena A, Dwyer AA, Peters S, Eicher M (2018). Acceptance of the advanced practice nurse in lung cancer role by healthcare professionals and patients: a qualitative exploration. J Nurs Scholarsh.

[43] Zhou J, Wang C (2020). Improving cancer survivors’ e-health literacy via online health communities (OHCs): a social support perspective. J Cancer Surviv.

[44] Sperisen N, Arditi C, Schaffar R, Dietrich PY, Rapiti E (2024). Experiences and needs of Swiss cancer survivors in the domains of health-related information and the healthcare system. Cancers (Basel).

[45] Rubinelli S, Purnat TD, Wilhelm E (2022). WHO competency framework for health authorities and institutions to manage infodemics: its development and features. Hum Resour Health.

[46] Zarocostas J (2020). How to fight an infodemic. The Lancet.

[47] De Gani SM, Berger FMP, Guggiari E, Jaks R (2022). Relation of corona-specific health literacy to use of and trust in information sources during the COVID-19 pandemic. BMC Public Health.

[48] Ribi K, Schmidt F, Haslbeck J (2025). Pilot testing a peer-led self-management program for women after breast cancer. Semin Oncol Nurs.

[49] Cuthbert CA, Farragher JF, Hemmelgarn BR, Ding Q, McKinnon GP, Cheung WY (2019). Self-management interventions for cancer survivors: a systematic review and evaluation of intervention content and theories. Psychooncology.

[50] Bana M, Ribi K, Kropf-Staub S (2019). Implementation of the Symptom Navi © Programme for cancer patients in the Swiss outpatient setting: a study protocol for a cluster randomised pilot study (Symptom Navi© Pilot Study). BMJ Open.

[51] Valenta S, Miaskowski C, Spirig R (2022). Randomized clinical trial to evaluate a cancer pain self-management intervention for outpatients. Asia Pac J Oncol Nurs.

[52] De Gani SM, Berger FMP, Langkafel P, Matusiewicz D (2021). Digitale Gesundheitskompetenz: Brauchen Wir Den Digitalen Führerschein [Book in German].

[53] Capelan M, Battisti NML, McLoughlin A (2017). The prevalence of unmet needs in 625 women living beyond a diagnosis of early breast cancer. Br J Cancer.

[54] Jolidon V, Eicher M, Peytremann-Bridevaux I, Arditi C (2024). Inequalities in patients’ experiences with cancer care: the role of economic and health literacy determinants. BMC Health Serv Res.

[55] (2025). Consensus recommendations for cancer survivorship care in switzerland. Aerzteverlag medinfo AG.

[56] Jeitani C, Van den Broucke S, Leemans C (2025). Interventions addressing health literacy in cancer care: a systematic review of reviews. Int J Environ Res Public Health.

[57] Schulz PJ, Lindahl B, Hartung U, Naslund U, Norberg M, Nordin S (2022). The right pick: does a self-assessment measurement tool correctly identify health care consumers with inadequate health literacy?. Patient Educ Couns.

[58] Pleasant A, Maish C, O’Leary C, Carmona R, Okan O, Bauer U, Levin-Zamir D, Pinheiro P, Sørensen K (2019). International Handbook of Health Literacy.

