# Supplementary material for: Digital and Navigational Health Literacy in Swiss Cancer Survivors Compared With the General Population: Cross-Sectional Questionnaire Study
Source: J Med Internet Res. 2026 May 25;28:e84228. doi: 10.2196/84228 (PMC13200775; doi:10.2196/84228)
Supplement: Multimedia Appendix 2 [file jmir-v28-e84228-s002.docx]

## Multimedia Appendix 2


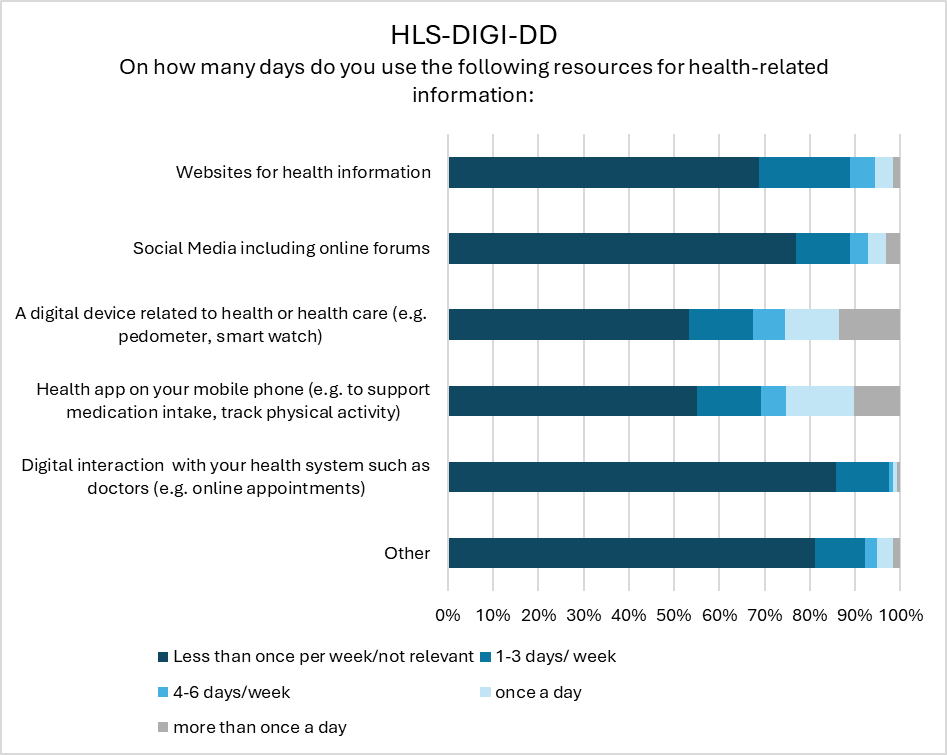


**Figure.** Frequency of use of digital health devices and resources (HLS_19_-DIGI-DD). Note that the response option “I don’t know” is not displayed since it is considered an invalid response.
