# Supplementary material for: Digital and Navigational Health Literacy in Swiss Cancer Survivors Compared With the General Population: Cross-Sectional Questionnaire Study
Source: J Med Internet Res. 2026 May 25;28:e84228. doi: 10.2196/84228 (PMC13200775; doi:10.2196/84228)
Supplement: Multimedia Appendix 3 [file jmir-v28-e84228-s003.docx]

## Multimedia Appendix 3

Differences in frequency of digital device use between cancer survivors and the general Swiss population (Mann-Whitney *U* Test)

|  | **Gender** | **Variable** | **Group** | **N** | **Mean rank** | **Sum of ranks** | **U** | **Z** | **p** |
| --- | --- | --- | --- | --- | --- | --- | --- | --- | --- |
| **SGP**  **whole group** | All | DIGI-DD mean value | SGP | 2526 | 1300.7 | 3285516.0 | 93915.0 | -8.81 | <.001 |
|  |  |  | CS | 127 | 1850.5 | 235015.0 |  |  |  |
|  | Men | DIGI-DD mean value | SGP | 1228 | 621.9 | 763736.5 | 9130.5 | -5.64 | <.001 |
|  |  |  | CS | 31 | 949.5 | 29433.5 |  |  |  |
|  | Women | DIGI-DD mean value | SGP | 1295 | 677.7 | 877624.5 | 38464.5 | -6.70 | <.001 |
|  |  |  | CS | 95 | 938.1 | 89120.5 |  |  |  |
| **SGP**  **sub-popu-lation** | All | DIGI-DD mean value | SGP | 930 | 502.9 | 467693.0 | 34778.0 | -8.22 | <.001 |
|  |  |  | CS | 127 | 720.2 | 91460.0 |  |  |  |
|  | Men | DIGI-DD mean value | SGP | 442 | 229.1 | 101246.5 | 3343.5 | -5.30 | <.001 |
|  |  |  | CS | 31 | 350.2 | 10854.5 |  |  |  |
|  | Women | DIGI-DD mean value | SGP | 487 | 273.6 | 133247.5 | 14419.5 | -6.27 | <.001 |
|  |  |  | CS | 95 | 383.2 | 36405.5 |  |  |  |

Note. The subpopulation comprises individuals with one or more chronic conditions; CS: cancer survivors; HLS: Health Literacy Survey; HLS_19_-DIGI-DD: Health Literacy Survey 2019-2021 – Digital Device subscale; U: Mann-Whitney-U-Test; SGP: Swiss General Population (Data provided by the Federal Office of Public Health)
