# Supplementary material for: Digital and Navigational Health Literacy in Swiss Cancer Survivors Compared With the General Population: Cross-Sectional Questionnaire Study
Source: J Med Internet Res. 2026 May 25;28:e84228. doi: 10.2196/84228 (PMC13200775; doi:10.2196/84228)
Supplement: Multimedia Appendix 4 [file jmir-v28-e84228-s004.docx]

## Multimedia Appendix 4

Regression analyses with all significant socio-demographic variables as independent and Health Education Impact Questionnaire (heiQ) subscales as dependent variables.

|  | **Predictor** | **B** | | **SE** | | **Beta** | | **T (df)** | | ***P*-value** | **95%-CI for B** | | **R^2^** |
| --- | --- | --- | --- | --- | --- | --- | --- | --- | --- | --- | --- | --- | --- |
|  |  |  |  |  |  |  |  |  |  |  | **Lower limit** | **Upper limit** |  |
| **heiQ 3**  **(emotional distress)** | Constant | 2.71 | 0.33 | |  | | 8.20 (112) | | <.001 | | 2.05 | 3.36 | .25 |
|  | ISCED 1 | -0.59 | 0.45 | | -0.11 | | -1.31 (112) | | .19 | | -1.49 | 0.30 |  |
|  | ISCED 5 | 0.23 | 0.13 | | 0.14 | | 1.78 (112) | | .08 | | -0.03 | 0.48 |  |
|  | Financial deprivation | -0.01 | 0.002 | | -0.34 | | -3.61 (112) | | <.001 | | -0.01 | -0.004 |  |
|  | Two or more chronic diseases | -0.002 | 0.18 | | -.001 | | -0.01 (112) | | .99 | | -0.35 | 0.35 |  |
|  | No chronic disease | 0.24 | 0.13 | | 0.17 | | 1.79 (112) | | .08 | | -0.02 | 0.49 |  |
|  | Social support | 0.03 | 0.03 | | 0.10 | | 1.07 (112) | | .29 | | -0.03 | 0.08 |  |
| **heiQ5**  **(constructive attitudes and approaches)** | Constant | 3.23 | 0.28 | |  | | 11.43 (112) | | <.001 | | 2.67 | 3.78 | .27 |
|  | ISCED 1 | -1.22 | 0.39 | | -0.27 | | -3.16 (112) | | .002 | | -1.99 | -0.46 |  |
|  | ISCED 7 | 0.23 | 0.12 | | 0.16 | | 1.93 (112) | | .06 | | -0.01 | 0.47 |  |
|  | Financial deprivation | -0.01 | .002 | | -0.29 | | -3.14 (112) | | .002 | | -0.01 | -.002 |  |
|  | Two or more chronic diseases | -0.05 | 0.15 | | -0.03 | | -.34 (112) | | .73 | | -0.36 | 0.25 |  |
|  | No chronic disease | 0.09 | 0.11 | | 0.08 | | .81 (112) | | .42 | | -0.13 | 0.32 |  |
|  | Social support | 0.02 | 0.02 | | 0.08 | | .90 (112) | | .37 | | -0.03 | 0.07 |  |

| **heiQ6**  **(skill and technique acquisition)** | Constant | 4.53 | 0.71 | |  | | 6.41 (112) | | <.001 | | 3.13 | 5.93 | | .32 |
| --- | --- | --- | --- | --- | --- | --- | --- | --- | --- | --- | --- | --- | --- | --- |
|  | ISCED 1 | -0.51 | 0.34 | | -0.12 | | -1.50 (112) | | .14 | | -1.19 | 0.16 | |  |
|  | Financial deprivation | -.004 | .002 | | -0.19 | | -2.15 (112) | | .03 | | -0.01 | <.001 | |  |
|  | No chronic disease | 0.15 | 0.09 | | 0.14 | | 1.67 (112) | | .10 | | -0.29 | 0.33 | |  |
|  | Healthcare professional | -0.16 | 0.10 | | -0.13 | | -1.60 (112) | | .11 | | -0.35 | 0.04 | |  |
|  | Social support | 0.08 | 0.02 | | 0.32 | | 3.64 (112) | | <.001 | | 0.04 | 0.12 | |  |
|  | Time since diagnosis | 0.02 | 0.01 | | 0.23 | | 2.92 (112) | | .004 | | 0.04 | 0.01 | |  |
| **heiQ7**  **(social integration and support)** | Constant | 1.75 | | 0.26 | |  | | 6.76 (112) | | <.001 | 1.24 | | 2.27 | .46 |
|  | Female | -0.11 | | 0.10 | | -0.08 | | -1.12 (112) | | .27 | -0.30 | | 0.08 |  |
|  | ISCED 1 | -0.99 | | 0.47 | | -0.15 | | -2.11 (112) | | .04 | -1.92 | | -0.06 |  |
|  | Financial deprivation | -.002 | | .002 | | -0.10 | | -1.20 (112) | | .23 | -0.01 | | .001 |  |
|  | Two or more chronic diseases | 0.01 | | 0.12 | | 0.01 | | .11 (112) | | .91 | -0.22 | | 0.24 |  |
|  | Social support | 0.15 | | 0.02 | | 0.57 | | 7.20 (112) | | <.001 | 0.11 | | 0.19 |  |

| **heiQ8**  **(health service navigation)** | Constant | 3.01 | 0.27 |  | 11.36 (110) | <.001 | 2.49 | 3.54 | .31 |
| --- | --- | --- | --- | --- | --- | --- | --- | --- | --- |
|  | Female | -0.09 | 0.10 | -0.08 | -0.99 (110) | .32 | -0.28 | 0.09 |  |
|  | Age ≥ 70 | 0.16 | 0.14 | 0.09 | 1.13 (110) | .26 | -0.12 | 0.45 |  |
|  | ISCED 1 | -0.88 | 0.45 | -0.16 | -1.99 (110) | .05 | -1.77 | -.002 |  |
|  | Financial deprivation | -.004 | 0.002 | -0.25 | -2.62 (110) | .01 | -0.01 | -.001 |  |
|  | Two or more chronic diseases | -0.09 | 0.13 | -0.07 | -.74 (110) | .46 | -0.34 | 0.16 |  |
|  | No chronic disease | 0.05 | 0.09 | 0.05 | 0.55 (110) | .59 | -0.13 | 0.24 |  |
|  | Social support | 0.06 | 0.02 | 0.26 | 2.86 (110) | .005 | 0.02 | 0.10 |  |

Note: Dependent variables: mean values of heiQ (health education impact questionnaire) scales; ISCED: International standard classification of education (8 level; 1=primary education, 8=doctorate)
